# The electromagnetic wave energy effect(s) in microwave–assisted organic syntheses (MAOS)

**DOI:** 10.1038/s41598-018-23465-5

**Published:** 2018-03-26

**Authors:** Satoshi Horikoshi, Tomoki Watanabe, Atsushi Narita, Yumiko Suzuki, Nick Serpone

**Affiliations:** 1 Department of Materials and Life Sciences, Faculty of Science and Technology, Sophia University, 7-1 Kioicho, Chiyodaku, Tokyo, 102-8554 Japan; 2 Microwave Science Research Center (MSRC), Sophia University, 7-1 Kioicho, Chiyodaku, Tokyo, 102-8554 Japan; 3 PhotoGreen Laboratory, Dipartimento di Chimica, Università di Pavia, Via Taramelli 12, Pavia, 27100 Italy

## Abstract

Organic reactions driven by microwaves have been subjected for several years to some enigmatic phenomenon referred to as the microwave effect, an effect often mentioned in microwave chemistry but seldom understood. We identify this microwave effect as an electromagnetic wave effect that influences many chemical reactions. In this article, we demonstrate its existence using three different types of microwave generators with dissimilar oscillation characteristics. We show that this effect is operative in photocatalyzed TiO_2_ reactions; it negatively influences electro-conductive catalyzed reactions, and yet has but a negligible effect on organic syntheses. The relationship between this electromagnetic wave effect and chemical reactions is elucidated from such energetic considerations as the photon energy and the reactions’ activation energies.

## Introduction

The German chemist Theodor Grotthuss was the first to formulate the first law of photochemistry in 1817; he postulated that a reaction could be driven by light when the energy of light is absorbed by molecules^1^. Grotthuss’ idea remained unknown for many years until John W. Draper, an expert in chemistry and photographic processes, proposed in 1842 that only absorbed light rays can produce chemical changes. For this reason, Grotthuss’ postulate is better known today as the Grotthuss-Draper law or the first law of photochemistry^2^. The photographic technique developed by Louis Jacques Mandé Daguerre in 1839 as a practical photographic method was to become the starting point of photochemical developments. In this regard, the first researcher to develop true photochemical concepts that distinguished between primary and secondary processes taking place in a chemical system under light absorption was Johannes Stark (1908), who defined the primary process as the immediate absorption of a photon by a molecule or an atom followed by secondary processes. In effect, the law states that in a photochemical process (such as a photochemical reaction) one photon that is absorbed by a molecule causes the main photochemical process. In some circumstances, a molecule having absorbed a photon initiates a process that may involve several other molecules. The Stark-Einstein law is the second law of photochemistry named after Johannes Stark and Albert Einstein. This law states that for each photon of light absorbed by a chemical system, no more than one molecule is activated for a photochemical reaction, as defined by the quantum yield^2^. These two laws have elevated photochemistry as an academic (science) discipline over the last one hundred years. In addition, because of advances in light sources and various devices (engineering), such materials and processes as photocatalysts, organic solar cells, photopolymerization, quantum dots, and photochromism (among others) are currently being applied in various other fields.

The next significant surge in chemistry is microwave chemistry wherein microwaves, which represent electromagnetic waves other than light, were introduced as a driving force in the chemical reaction domain in the late 1980s. Since then, thousands of articles have appeared that pertain to microwave-assisted organic syntheses. There are three characteristics in this chemistry when using microwaves. The first is the high heating efficiency caused by the energy of the microwaves that directly reach and are absorbed by the substance. The second is the selectivity with which a specific substrate is heated, while the third characteristic is the enhancement of chemical syntheses by the microwaves’ electromagnetic wave energy, often referred to as the microwave effect (or non-thermal effect). Together with the first and second efficient heat effects, this opens a new path in microwave chemistry (see *e.g*., ref.^3^). The phenomenon of the microwave effect (third characteristic) impacting chemical reactions has been summarized in much of the relevant literature (see *e.g*., ref.^4^). Many researchers have tackled the elucidation of its mechanism from both theoretical and experimental perspectives; the resulting analyses of the mechanism suggest either the presence or the absence of the microwave effect^5^. The reason why the microwave effect has not been clarified to anyone’s satisfaction is that the term ***microwave effect*** in microwave chemistry includes numerous factors. Accordingly, the microwave phenomenon, which is not yet well understood, is bundled by the word microwave effect and thus has failed to being classified properly.

Thermal energy can be exchanged instantaneously via several mechanisms such as dipolar polarization, ionic conduction, and the Maxwell-Wagner effect (among others) when a substance absorbs microwaves. Consequently, is the microwave involvement in chemical reactions an electromagnetic wave effect (non-thermal effect) or is it a temperature effect (thermal effect)? Experience has shown that it is difficult to separate between the two. Such problems do not occur in photochemistry. Moreover, microwaves (e.g., at a frequency of 2.45 GHz; wavelength = 12.24 cm) cause electromagnetic waves to be distributed unevenly in the sample, thereby resulting in a non-uniform heating of the sample^6^. Hence, it is necessary to have a proper appreciation of microwave engineering (e.g., fabrication of a microwave apparatus) and to pay close attention to sample size and microwave irradiation methods. Failure to do so can result in erroneous reporting of microwave effects originating from incorrect temperature measurements. Consequently, it is necessary to implement the following points in order to clarify the microwave effect: (i) elucidate the meaning of the microwave effect, (ii) establish an experimental system from which we can observe the microwave effect on the sample using electromagnetic waves and heat, and (iii) build an experimental system (microwave engineering) such that measurement errors of temperatures are avoided.

To achieve some of the above points, we have examined photocatalyzed reactions enhanced by microwaves for nearly two decades during which we demonstrated the microwave effect by simultaneously irradiating the metal-oxide photocatalyst TiO_2_ with UV light and microwave radiation in reactions taking place during wastewater treatments, something that could not and cannot be achieved by conventional heating^7^. As a non-thermal effect, the microwave effect in photocatalyzed reactions was demonstrated by establishing that the lifetime and utilization efficiency of electrons excited by UV light in the photocatalyst are enhanced by the microwave radiation^8^. Other researchers have also investigated such phenomena. For instance, Kishimoto and coworkers reported that an applied microwave field can enhance the photocatalytic reduction of bipyridinium ion using CdS quantum dots (QDs) via an acceleration of the electron transfer process^9^. Among our series of experimental investigations, we noticed that changing the microwave generator from a magnetron type to a semiconductor type changed the efficiency of the photocatalyzed reaction even though other microwave and reactor components remained the same (see below). We hypothesized that this difference was due to the difference in the quality of the microwaves generated from each generator and we further deduced that the microwave effect in chemical reactions can be investigated using different types of microwave generators.

In the present study, we examined the microwave effect in chemical reactions using three different types of microwave generators and making certain considerations. First, we hypothesized that the microwave effect results from the influence of microwaves as electromagnetic waves when the sample is irradiated and the microwave energy is changed into heat. The microwave effect is defined as an electromagnetic wave effect(s) on the chemical reactions. Next, the experiments involved some microwave engineering: for example, (**i**) carrying out measurements of microwave oscillation for each generator, (**ii**) examining the electromagnetic wave effect in a heterogeneous reaction involving a metal-oxide photocatalyst, (**iii**) examining the electromagnetic wave effect in a heterogeneous organic synthesis involving metal catalysts, and (**iv**) carrying out intramolecular reactions or organic syntheses in homogeneous media using solvents of different polarities.

## Results and Discussions

### Observation of microwave oscillation power for each generator

**Figure 1:**
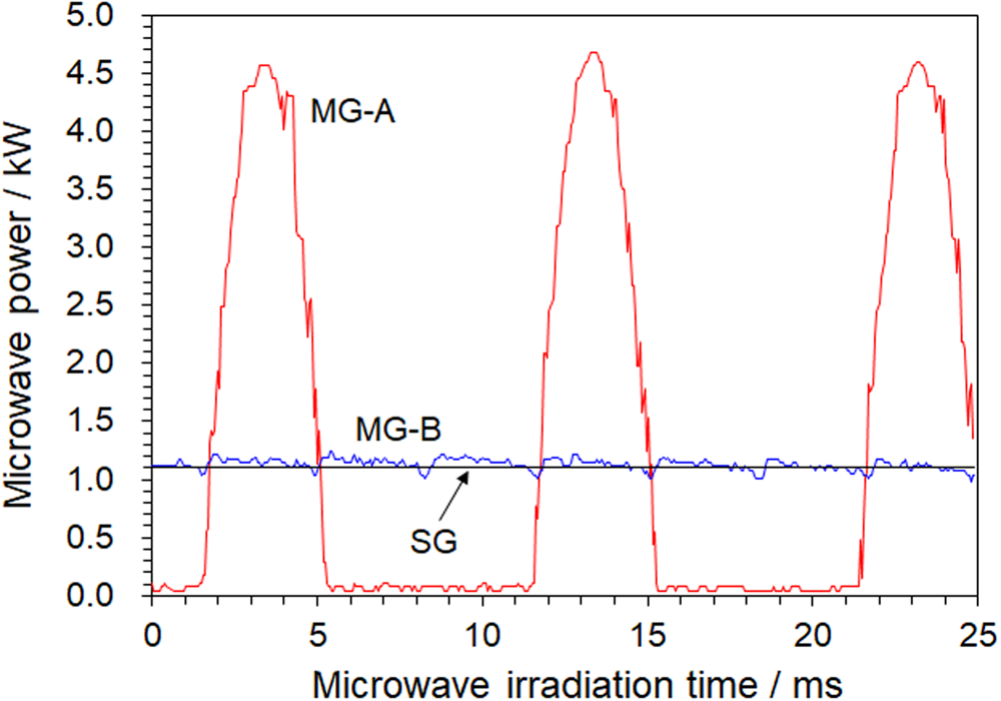
Measurement of microwave oscillation power in magnetron generator (MG-A: UM-1500SS-A system; MG-B: UM-1500IS-B system) and semiconductor generator (SG: M2A-R system).

The three different microwave generators used in our experiments were connected to a microwave irradiation device, with each generator continuously oscillating at a power of 1100 W; the incident microwave power was measured with a power sensor. The monitored microwave power transmitted at each oscillation cycle is displayed in Fig. 1. Pulsed irradiation was performed at intervals of about 5 ms (milliseconds) despite setting the irradiation power at 1100 W for the UM-1500SS-A generator (MG-A). The maximum oscillation power rose to 4672 W and then dropped to 0 W within about 5 milliseconds; when this waveform was averaged, it became ca. 1100 W. The UM-1500IS-B generator (MG-B) generated microwaves with an error of about ±110 W with respect to the set microwave power of 1100 W. The microwave irradiation power of the semiconductor-type generator (SG) continued to oscillate steadily at 1100 W in contrast to the MG-B generator. Thus, the MG-A instantaneously irradiates the sample with microwave power at a maximum average power about 4.2 times greater than those of the MG-B and SG generators. Most of the commercial devices used in the fields of microwave chemistry and materials science emit microwave pulses of several milliseconds in width similar to those from the MG-A generator. Note that the pulse width and amplitude depend on the power supplied and the performance of the magnetron. No doubt, the oscillation behavior of the microwaves caused by the difference in microwave generators likely influences a chemical reaction. Question is then why did chemists not notice such characteristics of the generators before? Generally, when a power meter that measures the oscillation power of microwaves is connected to a waveguide and irradiation with 1100 W is performed with the MG-A generator, the value shown is approximately 1100 W; however, pulsed oscillations are not shown. Many power meters cannot display the average value of the microwave irradiation power because the measurement intervals and the measurement accuracy are not high. In our experiments, we could observe it because we used a highly accurate power sensor used in communications applications and in a synchroscope system.

Such a difference in the characteristics of the generators may be the driving cause that influences a microwave-assisted chemical reaction. That is, the characteristics of the three generators are reminiscent of the following hypothesis. Since the period of oscillation of the molecule is from ca. 100 femtoseconds to 1 picosecond, the time that a molecule undergoes a chemical reaction, and a chemical bond is either broken or generated, is also about the same as the period of oscillation of the molecule, namely in the femtosecond to picosecond domains^10^. Question then is what is the timescale of the conversion of electromagnetic energy into heat? When dipolar polarization is the main phenomenon, dielectric heating involves unorganized movements at the microscale owing to the inability of molecular clusters to move in unison with the electric field. This hysteresis phenomenon explains how the organized energy of the electromagnetic field is transferred as a sort of Brownian movement in matter. The characteristic timescale of this conversion is some picoseconds^11^, i.e. very fast compared to thermal diffusion that occurs in seconds. On the other hand, many reactions of molecules in organic syntheses proceed in a timescale of milliseconds. The accumulated amount of microwave energy of the three generators is the same because it is averaged over minutes or hours. However, the microwave power from the MG-A system could be up to 4.2 times greater with respect to the MG-B and SG systems for the reaction time of one molecule. If the microwave effect proceeded directly from the microwave energy, we would anticipate the synthetic yields obtained from the chemical synthesis with the three generators to be different. In order to verify this hypothesis, a heterogeneous photocatalyzed reaction, a metallic solid catalyzed reaction, a model organic synthesis, and the heating behavior of solvents were examined next.

### The electromagnetic wave effect(s) in a TiO_2_ photocatalyzed reaction

**Figure 2:**
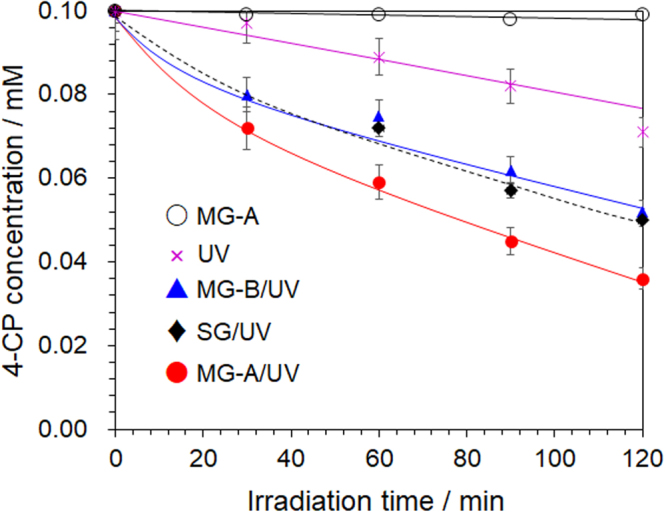
Time course of the decrease of 4-chlorophenol (4-CP) concentration using UV absorption loss at 279 nm in HPLC chromatograms during its degradation by microwave irradiation with the MG-A generator (MG-A) without TiO_2_, by the photocatalyzed oxidation in aqueous TiO_2_ dispersions (UV alone), and by an integrated UV-/microwave-assisted photocatalyzed degradation process in aqueous TiO_2_ dispersions with the MG-A (MG-A/UV) MG-B (MG-B/UV), and a SG (SG/UV) generators.

Loss of UV absorption intensity at 279 nm during the photodegradation of 4-chlorophenol (4-CP) in aqueous solutions and monitored by HPLC chromatography is shown in Fig. 2 as loss of concentration. The photodecomposition of 4-CP was carried out using the three microwave generators (MG-A, MG-B and SG) in combination with UV irradiation or with UV light alone. The heating rates and the reaction temperatures were otherwise the same (ca. 101 °C). Results show that the extents of photodegradation of 4-CP were 64% (MG-A/UV), 48% (MG-B/UV) and 50% (SG/UV) for an irradiation time of 120 min. Clearly, the UV-assisted TiO_2_ photocatalyzed reaction with the microwaves from the MG-A generator (MG-A/UV) showed a greater accelerating effect against the other two generators (MG-B/UV and SG/UV). Interestingly, in the UV method alone (conventional photocatalyzed reaction), the photodegradation rate of 4-CP was lower than the rates from any of the three microwave/UV methods. Moreover, no reaction occurred (within experimental error) when the metal-oxide was irradiated solely with the microwaves from the MG-A generator. The quantum energy of the microwave is low (about 1 × 10^−5^ eV), even though the MG-A generator delivered 4.2 times the microwave energy, so that it is impossible to affect the photocatalyst by the electromagnetic wave effect alone. Note that a quantum energy of 3.0‒3.2 eV is required to activate the metal oxide used. Therefore, although the energy source of photocatalyzed reactions is the UV light, the microwaves’ electromagnetic wave effect minimizes the recombination of excited electrons (i.e., conduction band electrons) with the photogenerated holes in this chemical reaction and so causes the rate of electron transfer to be enhanced. Evidently, microwaves contribute to the electronic events within the photocatalyst. No effect was observed when visible light and microwave radiation were used simultaneously^12^.

In summary, irradiating the metal oxide TiO_2_ with both UV light and microwaves enhanced the photocatalyzed reaction. However, this efficiency varied depending on the kinds of generators used. Despite the reaction temperature being in all cases ca. 101 °C and the microwave irradiation power nearly the same, the generator with the higher instantaneous peak intensity seems more advantageous under the UV/MW conditions used. Since the promoting effect of microwaves in the photocatalyzed reaction contributes to the behavior of electrons within the metal oxide, the instantaneous microwave power (electric field) has a more positive effect on electron transfer ***prior*** to the microwave energy being converted into heat in the metal oxide. Clearly, the electromagnetic wave effect has proven to be a non-insignificant actor in the photocatalyzed reaction.

### The electromagnetic wave effect(s) in chemical synthesis in the presence of a metallic solid catalyst

**Figure 3:**
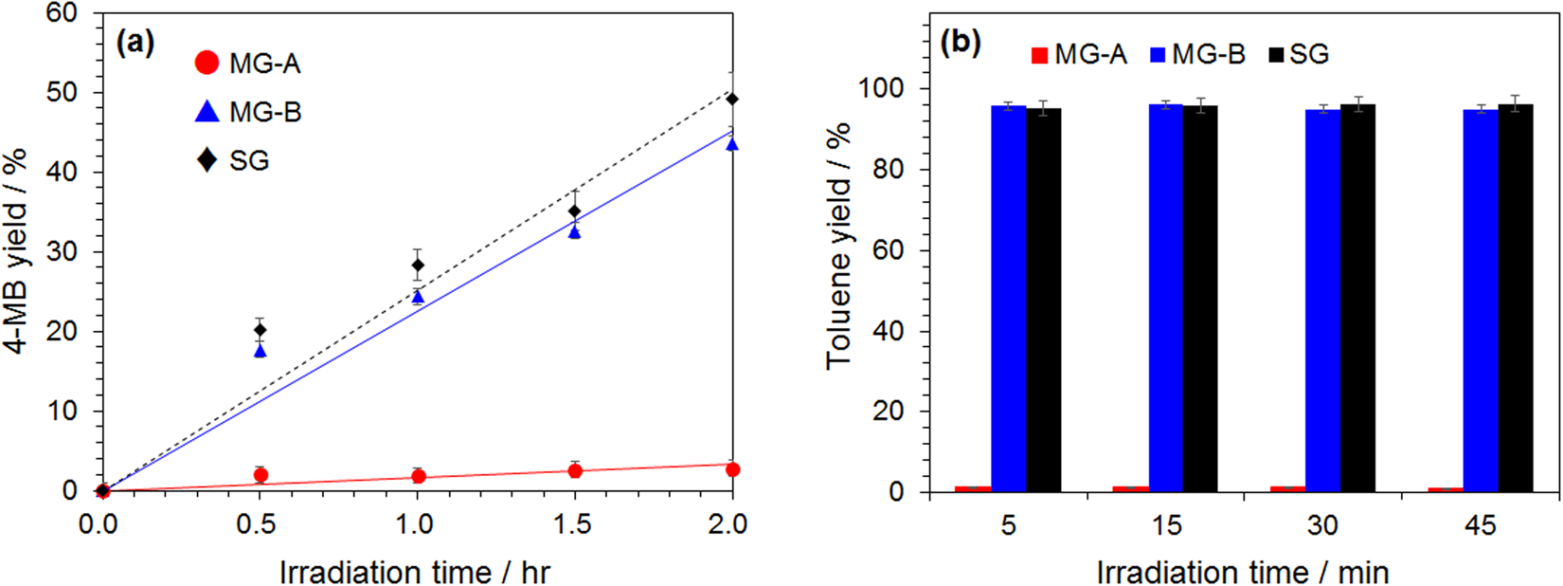
(**a**) Product yields of 4-methylbiphenyl (4-MB) with dispersed Pd/AC heterogeneous catalysts in toluene/1-hexamol solvent by microwave heating with three different microwave generators (MG-A, MG-B and SG; power was fixed at 51 W). (**b**) Evolution of hydrogen gas from the dehydrogenation of methylcyclohexane with dispersed Pt/AC heterogeneous catalysts by microwave heating with the three different microwave generators (power was fixed at 61 W).

4-Methylbiphenyl was synthesized by the Suzuki-Miyaura coupling reaction in the nonpolar solvent toluene in the presence of Pd/activated carbon (Pd/AC) catalyst particles (Fig. 3a). After microwave irradiation for 98–154 seconds with each generator (input power, 51 W), the reaction temperature reached the boiling point of toluene (ca. 110 °C). To the extent that the microwaves’ relative dielectric loss of toluene solvent is extremely low (*ε*_*r*_″ = 0.07), microwave heating does not occur via direct absorption of the microwaves by toluene; rather, the heating of toluene is indirectly caused by the microwave selective heating of the Pd/AC catalyst particulates. In the case of MG-B and SG generators, the synthesis yield of 4-methylbiphenyl increased in proportion to the synthesis time: yields of ca. 44% (MG-B) and ca. 49% (SG) were observed after 2 hours of irradiation. On the other hand, the synthesis yield with the MG-A system was only 2.0% by microwave irradiation for 30 min, and remained nearly so (ca. 2.8%) even after microwave irradiation for 2 hours thereafter. Clearly, the synthesis yield was significantly lower when using the MG-A generator; that is, although the total microwave irradiation power was the same for the reaction time of 2 hours, irradiation with pulsed microwaves of 5 ms duration had a negative influence on the synthesis of 4-methylbiphenyl.

We also conducted a continuous experiment involving the synthesis of toluene via the dehydrogenation of methylcyclohexane using Pt/activated carbon (Pt/AC) catalyst particulates as yet another model reaction (Fig. 3b). In this experiment, the Pt/AC catalyst particles were packed into a flow reaction vessel; methylcyclohexane was then continuously introduced into the reactor. The yield of toluene in the discharged solution was monitored continuously. When the MG-B or SG microwave generators were used, the rate of formation of toluene immediately after microwave irradiation was maintained at 95% or more. On the other hand, when the MG-A generator was used, the rate of toluene formation remained at 1.4%, and no rise in toluene yield was observed (see Fig. 3b).

Clearly, even though two kinds of syntheses were carried out using metallic solid catalysts, the behaviors of the generators displayed an otherwise identical trend, in that the yield was lowest when using the microwaves emitted by the MG-A generator. In addition, even though the electromagnetic wave effect did clearly manifest itself, the effect was not a positive one.

In an earlier study, we reported that microwave discharges (hot spots by local polarizations with the Maxwell-Wagner effect) occur on the metal catalyst surface because of the concentration of the microwaves’ electric field when carrying out a solid-liquid reaction in the presence of metallic catalyst particulates^13^. We hypothesized that the catalytic activity during the process was affected because the metal catalyst particles either melted or formed agglomerates because of heat from the hot spots^14^. The decrease of product yields when using the MG-A generator was deduced to be caused by the formation of hot spots on the catalyst surface, which we confirmed through observation with a high-speed camera. Indeed, formation of intense hot spots occurred within the reaction vessel during the synthesis of 4-methylbiphenyl and toluene (see, for example, Fig. S-1a). On the other hand, no occurrence of hot spots was observed when these reactions were carried out using the MG-B and SG generators.

An important aspect of the mechanism of formation of hot spots is the relationship between the size of the catalyst particulates and the space between the catalyst particles. With the catalyst sizes used in our experiment, when the space between the catalyst particulates was about 9 μm, the electric field of the microwaves was significantly concentrated within the space between the particles with the electric field intensity increasing more than tenfold thereby causing the discharge to occur^12^. Carbon-based solid materials are suitable for microwave heating because of the delocalized *π*-electrons from the *sp*2-hybridized carbon networks^15^. Transfer of free electrons on the carbon surface causes Joule heating of the carbonaceous material with the microwaves’ electric field^16,17^. Therefore, the movement of electrons on the activated carbon surface irradiated with microwaves is accelerated, which triggers the formation of hot spots. The travel time (electron current speed) of the electrons on the activated carbon surface irradiated with microwaves is less than milliseconds.

Continuing further verification experiments, the synthesis of 4-methylbiphenyl was also performed using the SG generator system with the microwave irradiation power being increased stepwise from 51 W to 214 W (i.e., a nearly 4.2-fold increase). This led to the occurrence of hot spots (observed with the high-speed camera) generated when the microwave irradiation power reached 162 W (see Fig. S-1b), which under the conditions used represents the threshold power of generating hot spots in this reaction system. Since the occurrence time of the discharge by the microwaves’ electric field is less than a millisecond^18^, the maximum peak power of ca. 214 W used for the MG-A system exceeded the threshold of hot spot generation, and thus caused the instantaneous formation of hot spots. This explains why the chemical yields of 4-methylbiphenyl and toluene were low when using the MG-A system. In our previous study, we reported a way that could hinder the formation of hot spots by using a semiconductor generator^19^. In that study, we deduced that the narrow microwave frequency range generated by the semiconductor generator hindered the formation of hot spots. For the magnetron generator, however, the cause is more likely due to irradiation with the generated pulsed microwaves at peak power (see Fig. 1).

### The electromagnetic wave effect(s) in intramolecular and intermolecular reactions in homogeneous media

**Figure 4:**
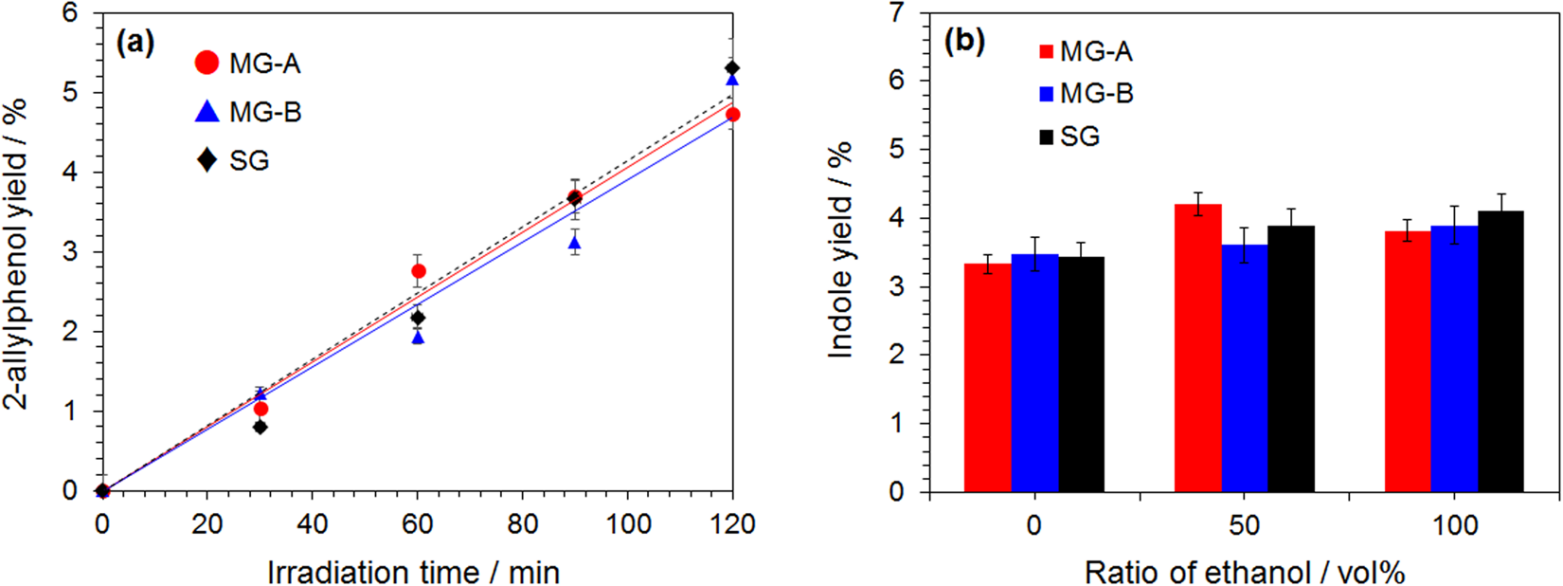
(**a**) Product yields of 2-allylphenol in dimethyl sulfoxide solvent by microwave heating with three different microwave generators (MG-A, MG-B and SG; power fixed at 60 W). (**b**) Product yields of indole in 100% ethanol solvent or 100% benzene solvent or in mixed benzene/ethanol solvents (50:50 vol.%) by microwave heating with three different microwave generators {MG-A, MG-B and SG; power, 53 W (ethanol), 161 W (mixed solvent), 921 W (benzene)}.

The microwave-assisted synthesis of 2-allylphenol was carried out by the Claisen rearrangement of allylphenyl ether in dimethyl sulfoxide solvent. Approximately 4 min after the start of heating, the sample temperature reached 180 °C, after which microwave heating and the heat released to the atmosphere (ca. 25 °C.) balanced and remained constant thereafter (see Fig. S-2). The difference in temperature rise in each generator was within 2 °C. The synthesis yields of 2-allylphenol as a function of irradiation time are shown in Fig. 4a. Within experimental error, no difference was observed in the yields of 2-allylphenol with respect to the three kinds of microwave generators used. In the metal-solid catalyzed reaction, the synthesis yield decreased when the MG-A generator was used; however, in the absence of the catalyst the intramolecular reaction was not affected by the momentary energy (power) increase of the incident microwaves.

The synthesis of indole was carried out under microwave irradiation in a solvent (3.00 mL) having different polarities with varying volume ratios of benzene and ethanol: the ratio of ethanol to benzene was 100:0, 50:50, and 0:100 (in vol.%). As the volume ratio of benzene increased, the heating efficiency of the microwaves decreased significantly (in fact extremely low). Results of measurements of the dielectric characteristics of each reaction component were obtained using a Keysight Technology Vector Network Analyzer (E5071C) with a Slim Form Probe Kit (Open-end coaxial probe; Option 030) and dielectric probe software application; calibration was performed before using a standard calibration kit and ultrapure water. The microwave relative dielectric loss factors (*ε*_*r*_″) and relative dielectric constants (*ε*_*r*_′) were 7.5067 and 6.6221 for ethanol, 0.0001 and 2.3300 for benzene, 1.5410 and 5.3116 for phenylhydrazine, 3.7592 and 7.2444 for pyruvic acid, and 0.0223 and 0.9050 for indole. The reaction temperature was 70 °C after 2 hours. The yields of indole for each of the solvents are reported in Fig. 4b. In the reaction system with high ethanol content, the ethanol was the heat source after absorbing the incident microwaves. Microwaves easily heated the mixed solution. On the other hand, when the solvent was 100% benzene, the heating efficiency of the mixed solution was very low. Note that the solution is currently homogeneous. That is, although it is the same chemical synthesis, it created different conditions of microwave absorption. The difference in product yields when using the MG-B and SG generators with respect to MG-A was ca. 14% (MG-A and MG-B in 50 vol.% ethanol); this difference was unlikely due to microwave pulsed irradiation, at least within experimental error. Earlier, we reported that the microwave heating efficiency depends on the state of the mixture, not on the mixing ratio, for a mixed solution of water and alcohol^20^. Therefore, this reaction system is associated with selective heating by microwaves at the cluster level.

The reason why there is a negligible difference in synthesis yields from the three different generators must be considered from the viewpoint of the reactions’ activation energies. The theoretical activation energy required for the synthesis of 2-allylphenol from 2-allylphenyl ether is 134 kJ mol^−1^^21^. Hence, the activation energy required for the synthesis of 1 molecule of 2-allylphenol is 2.226 × 10^−18^ J. One photon of the electromagnetic wave energy from the 2.45-GHz microwaves is 1.623 × 10^−24^ J {*E = *h*v = *(6.626 × 10^−34^ J s) × (2.45 × 10^9^ s^−1^)}. Accordingly, attempts to synthesize 2-allylphenol only with microwave energy will require irradiating the molecule with 7.29 × 10^7^ photons. That is, in order for the microwaves to cause some changes in the substance, energy must be supplied to the substance repetitively over a period of time (multi-photon absorption). For a quantum yield of 1, this would necessitate the accumulation of energy from the 7.29 × 10^5^ photons. However, multi-photon processes tend to be very inefficient, even with intense laser light, and so such multi-photon processes involving microwave radiation are unlikely to be of any significance. Moreover, the photon energy corresponding to the 2.45-GHz microwaves is about 1 × 10^−5^ eV. Electronic excitation and chemical reactions implicating atoms typically occur in the energy region of ca. 10 eV or more. Since microwaves are electromagnetic waves, energy confinement and integration are possible by using a cavity resonator. However, if there is a specimen in the cavity, the microwaves are changed to heat within the relaxation time of the molecules the moment the microwaves are absorbed by the specimen (on the order of several picoseconds)^22,23^. Therefore, it is not possible to accumulate quanta of energy in the process of irradiating the dielectric substrate with microwaves and use it for the reaction. On the other hand, what about the heat accumulated from the microwaves? The microwave energy is irreversibly dissipated into thermal energy when absorbed by the dielectric sample. To the extent that the rate of heat generation in the dielectric sample exceeds the rate of heat radiated to the atmosphere, the heat accumulates in the dielectric sample, thereby allowing the chemical reaction to proceed via this phenomenon. As a further hypothesis, heating samples by irradiating with pulsed microwaves would not lead to any further accelerating effect on the reaction with additional microwave energy. In other words, if heating proceeded by pulsed microwave irradiation, the heat would then be released to the atmosphere at the next moment.

**Figure 5:**
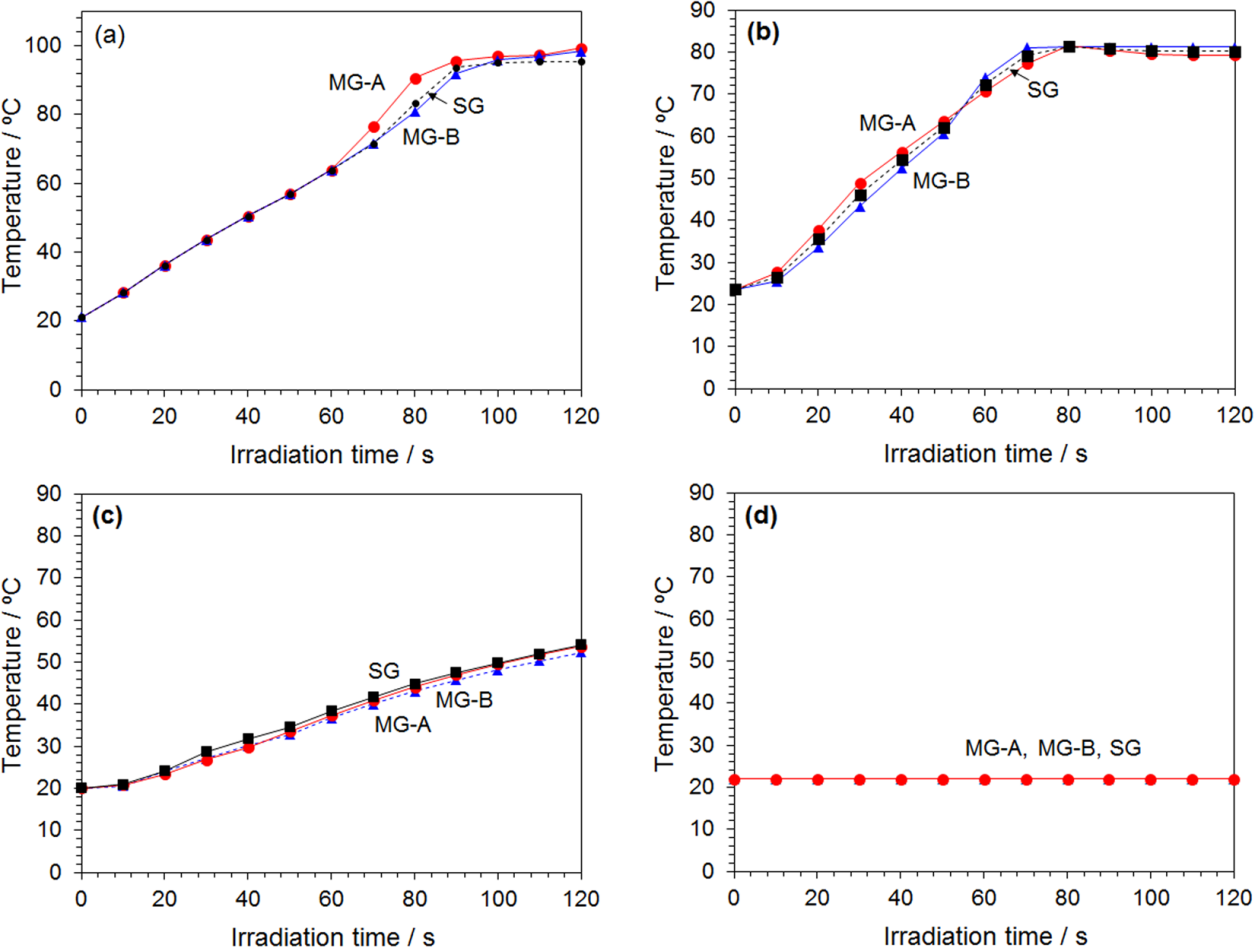
Temporal changes in temperature rise for (**a**) ion-exchanged water, (**b**) ethanol, **(c**) mixed ethanol/benzene (1:1 vol.%) and (**d**) benzene using the microwave traveling waves from the UM-1500SS-A (MG-A), UM-1500IS-B (MG-B) and M2A-R (SG) microwave generators.

To verify the above inferences, we examined how the temperature would vary for solvents composed of ion-exchanged water, ethanol (polar organic solvent), benzene (nonpolar organic solvent), and ethanol/benzene (5.00 mL each) placed in a quartz reactor. The microwave device used for this purpose is illustrated below in the experimental section (Section 4); the continuous microwave power was set at 50 W for all the microwave generators. The temperature of each solution was measured at 5-second intervals with the optical fiber tip positioned at the center of the solution. During the experiment, we confirmed that there was zero reflection of the microwaves as monitored by the power sensor. The heating rate for heating the ion-exchanged water (electric conductivity < 0.1 mS m^−1^) subjected to microwaves from each generator is displayed in Fig. 5a, which reveals that the difference in temperature rise was below 4%. Next, we compared the heating rates for ethanol (Fig. 5b), for the 1:1 mixed solvent ethanol/benzene (Fig. 5c), and benzene (Fig. 5d). No differences were observed in the rates of temperature rise in the organic polar solvent, nonpolar solvent and the mixed polar/non-polar solvent mixture. The overall results show that the total irradiation energy of the microwaves is a determinant in the temperature rise in the solvents examined; that is, there was no change when the solvents were dielectrically heated with continuous microwave irradiation or when irradiating with the 5-ms pulsed microwaves. Since the total energy did not change even when the irradiation conditions of the microwaves changed, the heating efficiency was not affected and consequently the chemical reactions should not be affected.

## Concluding remarks

The electromagnetic wave effect (i.e. the non-thermal effect) of microwaves has been demonstrated to be a pertinent component in some chemical reactions. For instance, it affects electron transfer in organic materials owing to the microwaves’ electric field. In particular, it affects chemical reactions where electrons are the principal factors, reminiscent of photochemical organic reactions. However, because the quantum energy of the microwaves is low (ca. 1 × 10^−5^ eV), it is necessary to integrate the electromagnetic wave energy. To the extent that this is not possible, however, the electromagnetic wave effect may not directly affect many organic reactions. Moreover, in an organic synthesis in which the reaction is driven by thermal energy (heat), even when the energy of the microwaves is added as an electromagnetic wave, it did not support or enhance the thermal reaction. Note that there may be exceptions to this inference as the number of organic reactions so far examined is rather limited. How then can one use the electromagnetic wave effect(s) in chemical reactions to make use of some of its advantages? This electromagnetic wave effect appears in chemical reactions particularly when electrons are directly involved in the reactions. For instance, the electromagnetic wave effect will likely appear when the chemical reaction progresses via electron transfer such as those that may implicate a photocatalyst. In such a case, the influence of microwaves (as electromagnetic waves) would contribute to chemical reactions. However, where a photocatalyst is involved, it will require its activation by UV light first and then subsequently aided by the microwaves. Consequently, the role of the electromagnetic wave effect in organic syntheses is to assist chemical reactions that are being driven by other forms of energies: for example, UV light. The role of this effect can then be construed as being an *electromagnetic wave catalyst*. Another influence of the electromagnetic wave effect on a highly conductive catalyst may be deleterious when such discharges as hot spots are formed. Nonetheless, when such discharges are controlled, they can be beneficial in chemical reactions involving conductive catalysts. Finally, having elucidated this effect of microwaves, utilization of microwaves should promote industrial development in such chemical fields as photochemistry.

## Experimental setup

### Outline of the microwave device for power and temperature measurements, and for chemical synthesis

**Figure 6:**
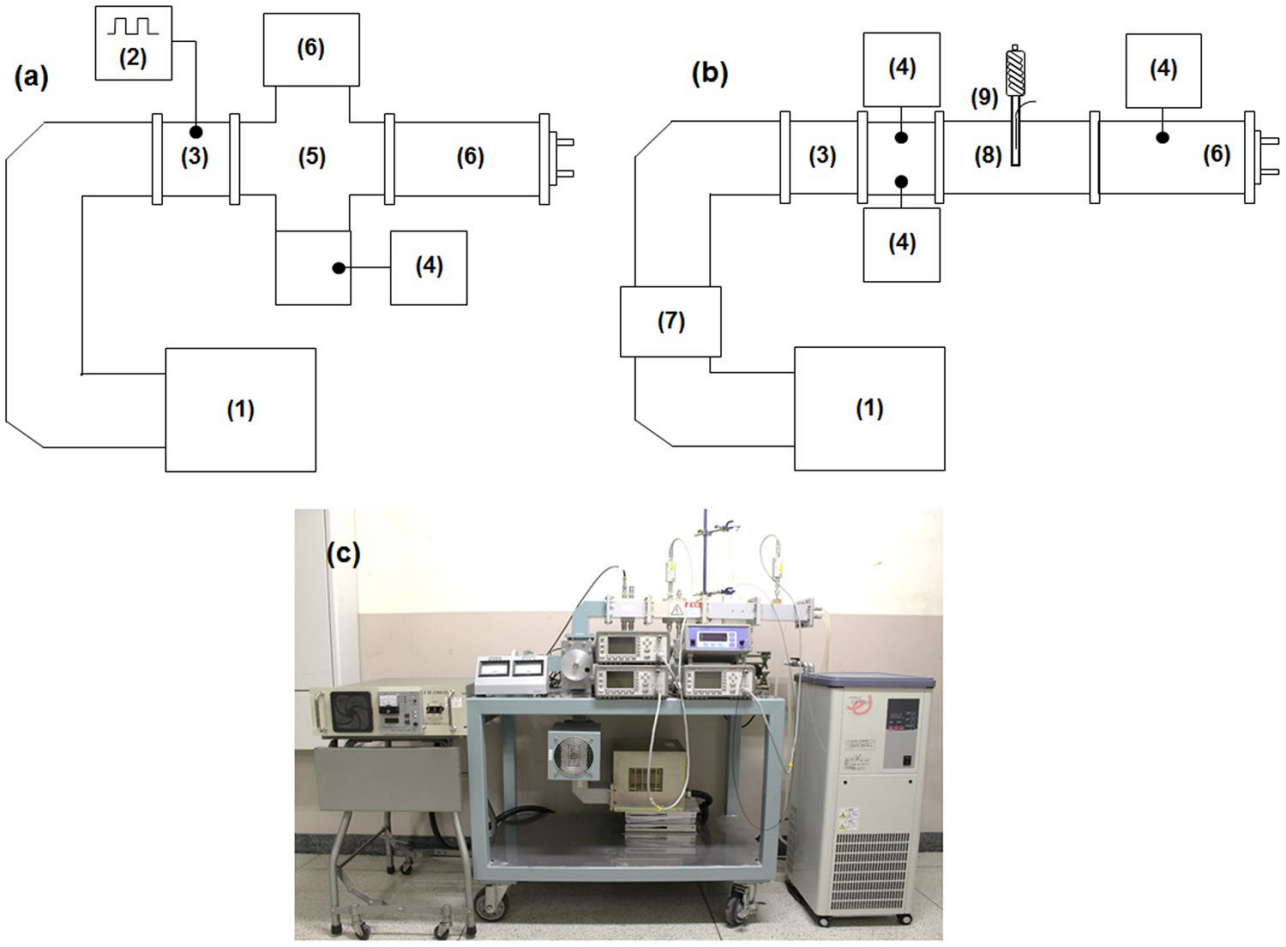
(**a**) Illustration of the microwave power measuring system; (**b**) illustration and (**c**) photograph of the microwave heating system: (1) microwave power generator (UM-1500SS-A: MG-A; UM-1500IS-B: MG-B, M2A-R: SG), (2) synchroscope MSO-X 3014 A, (3) power monitor (PM5000), (4) single channel power meter E4418B and Power sensor 8482 A, (5) cross type directional coupler, (6) dummy load, (7) isolator, (8) applicator and (9) sample reactor, reflux condenser and fiber optic thermometer.

The schematics and a photograph of the microwave device used in the experiments are shown in Fig. 6. The microwave generator utilized a magnetron-type microwave generator system {Micro Denshi Co., Ltd., UM-1500SS-A (abbreviated MG-A); maximum output power, 1500 W; power from an AC transformer type system}. This generator utilizes a power supply similar to the microwave generator system used in a general microwave chemical reaction apparatus. A microelectronic magnetron generator {Micro Denshi Co., Ltd., UM-1500IS-B (abbreviated MG-B); maximum output power, 1500 W; power from an inverter type system} was also used, which is a generator for plasma generation. Also used was a semiconductor generator system {Ampleon M2A-R (abbreviated SG): maximum output power, 1200 W}. Each generator was connected to a waveguide (WRJ-2), in turn connected to each instrument. The incident microwaves were absorbed with a dummy load; under the conditions used, none of the microwaves were reflected. The system schematically shown in Fig. 6a was used to measure the microwave power. The irradiated waveforms of the microwaves from the generators were monitored by the Synchroscope MSO-X 3014 A. A water-cooled dummy load was connected to the tip of a cross type directional coupler. The experimental setup to perform the chemical reactions and measure the temperatures of the solutions is shown in Fig. 6b and c. The waveguide was connected to the generator and to the sample heating applicator through an isolator, and to the incident and reflection sensors. A water-cooled dummy load was connected to the tip of the applicator, and the sample was subsequently irradiated with a traveling wave without resonating the microwaves. The reason for this was to prevent non-uniform temperature at the sample owing to the unevenness of the electromagnetic wave at the sample position originating from resonance conditions. Also, a power sensor was connected between the sample and the dummy load to monitor the power of the microwave passing through it. Continuous microwave oscillation was performed using a dedicated controller. The sample solution was introduced into a branched test tube made of quartz (inside diameter, 12 mm); a reflux tube was connected to the top of the test tube, and the temperature was measured using a fiber optic thermometer (Anritsu Meter Co., Ltd.). In preliminary experiments, we confirmed that the difference in temperature with respect to the vertical direction of the solution due to microwave irradiation was less than 1 °C. It was also confirmed during the experiment that reflection of microwaves was zero as per the power sensor. Note that the semiconductor generator was connected using a coaxial waveguide converter, and the microwave power loss that occurred was corrected in advance.

### Photocatalyzed degradation of 4-chlorophenol (4-CP)

Continuous microwave irradiation of the TiO_2_ dispersions was attained using the apparatus schematically displayed in Fig. 6b. The microwave power of the magnetron was fixed at 89 W. An air-equilibrated aqueous 4-CP solution (6 mL; 0.10 mM; initial pH 6.2) containing the photocatalyst particles (loading, 4 mg) was introduced into the quartz reactor/reflux condenser combination, after which the reactor was positioned in the waveguide. The UV irradiation source was a Toshiba 75 W low-pressure Hg lamp (irradiance, ca. 0.40 mW cm^−2^). UV light irradiation was performed using a fiber optic light-guide from the top of the reactor. Four different methodologies were examined to achieve the decomposition of the 4-CP. The first was the photocatalyzed degradation of 4-CP/TiO_2_ under UV light and microwave irradiation with the three microwave generators (MG-A/UV, MG-B/UV and SG/UV methods). The maximal temperature was 101 °C. The second method entailed the photocatalyzed degradation of 4-CP/TiO_2_ dispersions by UV irradiation alone (UV) as a conventional photocatalyzed reaction. The last method involved irradiation of the 4-CP/TiO_2_ dispersion with microwaves only (MW). The time profiles of the degradation ratio of 4-CP were obtained by monitoring concentration changes using a JASCO liquid chromatograph (HPLC) that was equipped with a JASCO UV-2070 UV-vis diode array multi-wavelength detector and a JASCO Crestpak C-18S column; the eluent consisted of a mixed solution of CH_3_CN and H_2_O (1:4, v/v ratio) and 1.7 vol.% of H_3_PO_4_ (pH 3.3).

### Synthesis of 4-methylbiphenyl

The synthesis of 4-methylbiphenyl by the Suzuki Miyaura cross-coupling process (Fig. S-3) was carried out to compare the results with our earlier study^24^. Microwave power was fixed at 51 W for each generator. The Pd/AC catalyst (0.18 g; mesh size of AC support, 0.95 mm; AC refers to the activated carbon particles), phenylboronic acid (0.96 mmol; 0.12 g), 1-bromo-4-methylbenzene (0.72 mmol; 0.12 g), K_2_CO_3_ as the base (1.40 mmol; 0.20 g), and the toluene/1-hexanol mixed solvent (6 mL; volume ratio, 1:1) were mixed and subsequently added to the cylindrical reactor under an Ar atmosphere. The relative dielectric loss factor of a sample solution without the Pd/AC catalyst particles was *ε*_r_″ = 0.51 at 25 °C, by comparison significantly smaller than that of pure distilled water (*ε*_r_″ = 8.7 at 25 °C); the toluene/1-hexanol solvent was slightly heated by the microwave radiation. The quartz cylindrical reactor was fitted with a reflux condenser (Fig. 6b). The Pd/AC catalyst was prepared using the procedure reported in our earlier study^24^. The quantity of Pd on the activated carbon particulates support was ca. 1.5 wt.% Pd ascertained by atomic emission spectroscopy using the Shimadzu ICPE-9000 apparatus. Reaction yields of 4-methylbiphenyl were determined by gas chromatographic analyses (Shimadzu model 2014 equipped with a Shimadzu GLC Ultra alloy-1 capillary column; helium was the carrier gas; column temperature, 100–260 °C; heating rate, 20 °C min^−1^) from samples suitably prepared from the various dispersions. A pure sample of 4-methylbiphenyl (Wako Pure Chemical Industries, Ltd., 100% GC standard) was used to calibrate the chromatograph. Generation of hot spots on the heterogeneous Pd/AC catalyst particles was monitored through the hole on the microwave cavity side using a Casio high speed camera (EX-ZR1000).

### Synthesis of toluene

The synthesis of toluene from the dehydrogenation of methylcyclohexane (MCH; Fig. S-4) was carried out to compare the results with our earlier study^25^. Microwave power was fixed at 61 W for each generator. The Pt catalyst supported on an activated carbon support (Pt/AC particulates; particle diameter: ca. 1.0 mm) was loaded (2.50 g) into a single-pass quartz fixed-bed tubular reactor (diameter: 1.7 mm; length: 55 mm) installed such that the MCH could penetrate from the bottom to the top of the waveguide. Moreover, the catalyst layer was located in the reaction vessel (see Fig. 6b) so as to be at the center of the waveguide. The upper and bottom parts of the reactor were closed with glass fiber wool to prevent loss of the Pt/AC catalyst powder introduced into the vertical reactor at the upper part of the tube. The temperature of the Pt/AC catalyst particulates at the inner reactor surface was measured by thermography (TVS-500, Nippon Avionics Co., Ltd.). The starting material methylcyclohexane (Wako Pure Chem. Ind., Ltd) was maintained at ambient temperature prior to being fed from the bottom part of the single-pass continuous flow reactor using a syringe pump (Isis Co. Ltd., Fusion 100; 0.10 mL min^−1^). The liquid organic hydride (MCH) was rapidly vaporized by the heated catalyst particulates subjected to microwave radiation. As a non-polar material, MCH is a poor absorber of microwaves (dielectric loss *ε*_r_″ = 0.01 at 25 °C), and thus was not directly heated by the microwave radiation. The discharged gases were automatically analyzed with an Agilent 490 micro-GC gas chromatograph using standard samples of MCH and toluene.

### Synthesis of 2-allylphenol

The microwave-assisted synthesis of 2-allylphenol was carried out by the Claisen rearrangement of allylphenyl ether in dimethyl sulfoxide solvent (Fig. S-5). The reactants were introduced in a quartz cylindrical reactor positioned in the applicator (Fig. 6b). Allylphenyl ether (1.00 mM) was added to dimethyl sulfoxide (1.00 mL) solvent and microwave heating was carried out at 180 °C using continuous irradiation with 60-W microwaves. After the reaction, the product was washed with 3.00 mL of ethyl acetate; the yield was calculated from the gas chromatographic data using a standard 2-allylphenol for calibration purposes.

### Synthesis of indole

Pyruvic acid (0.30 mM), phenylhydrazine (0.30 mM) and zinc chloride (0.30 mM) as the catalyst were added to a quartz cylindrical reactor equipped with a reflux condenser (Fig. 6b); solvents used (3.00 mL) had different polarities: ethanol solvent, benzene solvent and mixed ethanol/benzene (1:1 vol. %). The indole synthesis (Fig. S-6) was performed under microwave irradiation (power, 53 W for ethanol solvent; 161 W for the mixed solvent mixture; 921 W for benzene). The temperature was 70 °C for 2 hrs of irradiation. After the reaction, the product was washed with 3.00 mL of ethyl acetate; the yield was calculated from the gas chromatographic data.

## Electronic supplementary material

Supplementary figures
