# Supplementary material for: The electromagnetic wave energy effect(s) in microwave–assisted organic syntheses (MAOS)
Source: Sci Rep. 2018 Mar 26;8:5151. doi: 10.1038/s41598-018-23465-5 (PMC5980088; doi:10.1038/s41598-018-23465-5)
Supplement: Supplementary file 1 — Supplementary figures [file 41598_2018_23465_MOESM1_ESM.pdf]

The electromagnetic wave energy effect(s) in microwave–assisted organic syntheses (MAOS)

Satoshi Horikoshi,[1,2] Tomoki Watanabe,[1] Atsushi Narita,[1] Yumiko Suzuki,[1,2] Nick Serpone [3]

[1] *Department of Materials and Life Sciences, Faculty of Science and Technology, Sophia University, 7-1 Kioicho, Chiyodaku, Tokyo 102-8554, Japan*

[2] *Microwave Science Research Center (MSRC), Sophia University, 7-1 Kioicho, Chiyodaku, Tokyo 102-8554, Japan*.

[3] *PhotoGreen Laboratory, Dipartimento di Chimica, Università di Pavia, Via Taramelli 12, Pavia 27100, Italia.*

* Corresponding author to S.H.: (e-mail: horikosi@sophia.ac.jp)

Supplementary Figures

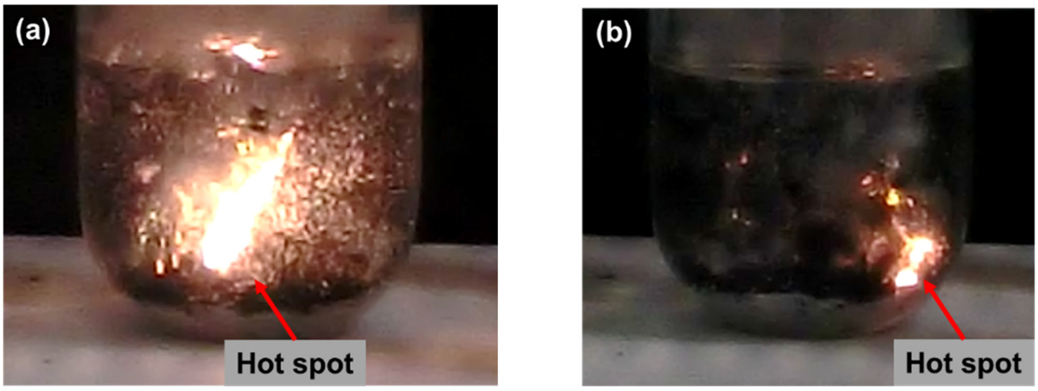

Fig. S-1. High-speed camera photograph of the electrical discharge occurring on (a) dispersed Pd/AC catalyst surface in 4-methylbiphenyl synthesis under microwave irradiation at 61 W with a GM-A generator system and (b) under microwave irradiation at 162 W with a SG generator system.

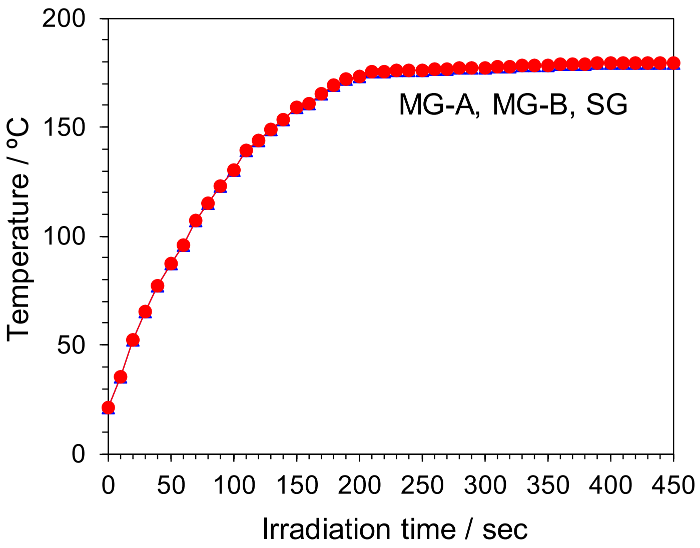

Fig. S-2. Temperature change in the synthesis of 2-allylphenol from allylphenyl ether in dimethyl sulfoxide solvent.

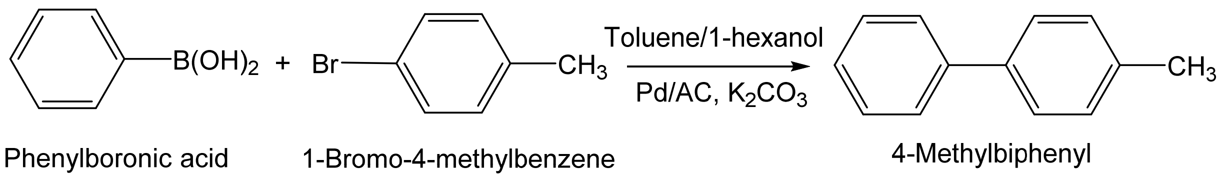

Fig. S-3 Synthesis of 4-methylbiphenyl with phenylboronic acid and 1-bromo-4-methylbenzene by the Suzuki Miyaura cross-coupling process with Pd/AC and K_2CO_3 base in toluene/1-hexanol solvent.

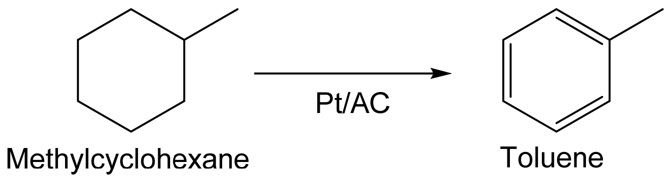

Fig. S-4 Synthesis of toluene from the dehydrogenation of methylcyclohexane with Pt/AC catalyst powder.

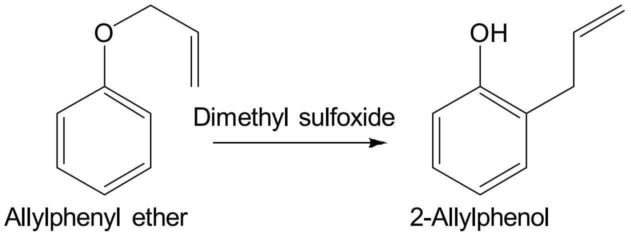

Fig. S-5 Synthesis of 2-allylphenol by Claisen rearrangement of allylphenyl ether in dimethyl sulfoxide solvent.

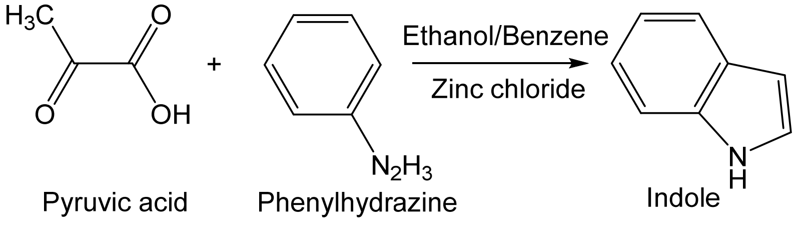

Fig. S-6 Synthesis of pyruvic acid, phenylhydrazine and zinc chloride in ethanol/benzene solvent.
